# The role of the hippocampus in generalizing configural relationships

**DOI:** 10.1002/hipo.22688

**Published:** 2017-01-24

**Authors:** Sam C. Berens, Chris M. Bird

**Affiliations:** ^1^ School of PsychologyUniversity of SussexFalmerBN1 9QHUnited Kingdom

**Keywords:** learning, memory, pattern completion, fMRI

## Abstract

The hippocampus has been implicated in integrating information across separate events in support of mnemonic generalizations. These generalizations may be underpinned by processes at both encoding (linking similar information across events) and retrieval (“on‐the‐fly” generalization). However, the relative contribution of the hippocampus to encoding‐ and retrieval‐based generalizations is poorly understood. Using fMRI in humans, we investigated the hippocampal role in gradually learning a set of spatial discriminations and subsequently generalizing them in an acquired equivalence task. We found a highly significant correlation between individuals’ performance on a generalization test and hippocampal activity during the test, providing evidence that hippocampal processes support on‐the‐fly generalizations at retrieval. Within the same hippocampal region there was also a correlation between activity during the final stage of learning (when all associations had been learnt but no generalization was required) and subsequent generalization performance. We suggest that the hippocampus spontaneously retrieves prior events that share overlapping features with the current event. This process may also support the creation of generalized representations during encoding. These findings are supportive of the view that the hippocampus contributes to both encoding‐ and retrieval‐based generalization via the same basic mechanism; retrieval of similar events sharing common features. © 2016 The Authors Hippocampus Published by Wiley Periodicals, Inc.

Learning relationships between features within the environment is central to complex behaviors such as navigation (Cohen and Eichenbaum, 1993). However, it is often not sufficient to rely on information that was learnt within a single episode. Frequently, memories acquired across multiple episodes must be integrated to allow the expression of novel behaviors (Bunsey and Eichenbaum, 1996). Such “memory generalizations” are also fundamental to the formation of the context‐free memory structures that characterize semantic knowledge (Eichenbaum, 2004).

The hippocampus has long been implicated in supporting memory generalization (Bunsey and Eichenbaum, 1996). Broadly speaking, there are two classes of model that attempt to describe the role of the hippocampus in generalization. Encoding‐based models propose that when related events are encoded, their representations overlap with each other and therefore contain information that is common to all of the events (Shohamy and Wagner, 2008). This results in unitary (or generalized) memory traces where the retrieval of any one detail automatically cues the retrieval of related features. In contrast, retrieval‐based models pose that study events are strictly “pattern separated” at encoding. Nonetheless, these models suggest that the hippocampus can engage “on‐the‐fly” generalization where representational overlaps are inferred during retrieval (e.g., Kumaran and McClelland, 2012).

Previous investigations have provided evidence for both encoding‐ and retrieval‐based accounts of hippocampal generalization (e.g., Shohamy and Wagner, 2008; Preston et al., 2004). Indeed, it has been suggested that these models are not mutually exclusive. In particular, the hippocampus may initially form pattern separated representations which gradually become integrated over a period of memory consolidation (Zeithamova et al., 2012b). Consolidation is associated with memory traces becoming more dependent on neocortical regions that underlie semantic memory (McClelland et al., 1995). This raises the possibility that generalization may sometimes depend on functions of the neocortex rather than the hippocampus.

To date, two studies have examined how the hippocampus and neocortex contribute to generalization over the course of both an initial learning phase and a generalization test (Zeithamova and Preston, 2010; Zeithamova, et al., 2012a). Both have provided good evidence for a mixed encoding/retrieval account. These studies used “relational inference” paradigms where hierarchical or pairwise relationships between stimuli must be inferred from training on a set of “premise pairs.” This contrasts with simpler forms of generalization where novel discriminations are made on the basis that stimuli belong to particular “equivalence classes.” For example, if particular configurations of stimuli are always rewarded, then they belong to the same superordinate class of rewarded items. It remains unclear whether or not generalizations reliant on equivalence class membership depend on hippocampal and/or neocortical involvement at either study or test. We used fMRI to examine the brain mechanisms involved in gradually learning and subsequently generalizing a set of visual discriminations. In particular, we explored whether hippocampal and/or neocortical activity at encoding, retrieval, or both, was associated with generalizations between equivalence classes.

Twenty‐three right‐handed students were recruited by way of online advertisement. All gave written informed consent to take part and were compensated for their time. Subjects had either normal or corrected‐to‐normal vision and reported no history of neurological or psychiatric illness. Of those who participated, eight did not exhibit sufficient levels of learning on the task to be included in the analyses (see below). As such, analyses reported here used data from 15 subjects (7 male) with a mean age of 23.8 years (SD = 4.14). The study was approved by the Brighton and Sussex Medical school's Research Governance and Ethics Committee.

During scanning participants learned a set of visual discriminations via trial‐and‐error (learning phase), and were subsequently tested on their ability to generalize what they had learned (generalization phase). Both learning and generalization occurred within a single scanning session and took place in the context of a first‐person virtual reality environment (see Fig. 1). On each trial, a scene was presented depicting two buildings positioned equidistantly from a start location. One building concealed a pile of gold (the “reward”). The location of this gold was determined by the configuration of wall textures rendered onto the towers of each building. Participants were required to select the rewarded building (within 3 s). Following a response, video feedback was presented (7 s) before an inter‐trial interval (1 s). The stimuli for the task were generated in Unreal Development Kit (Epic Games, 2012) and presented via the Cogent 2000 toolbox in MATLAB (Mathworks).

**Figure 1:**
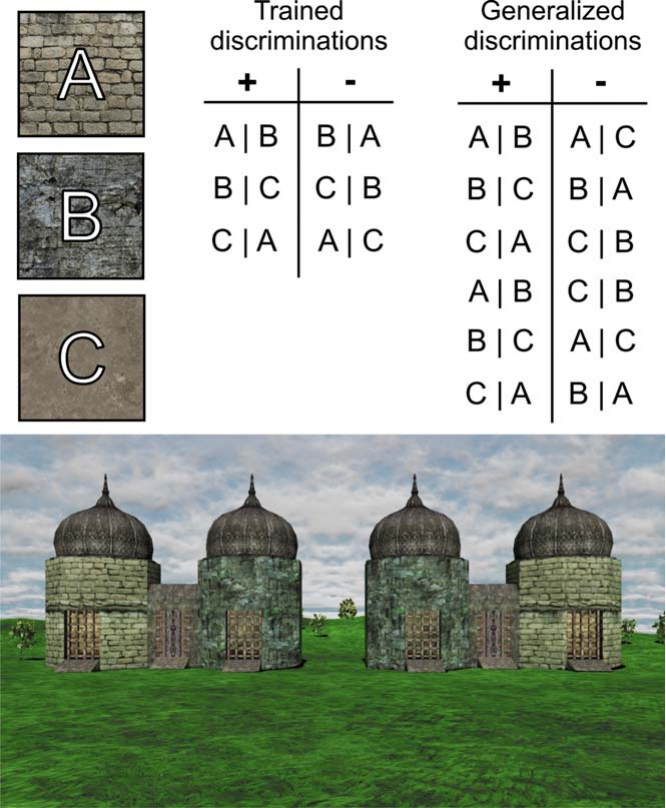
Upper: Details of the trained and generalized discriminations. Each capitalised letter refers to a unique wall texture as indicated by the three wall texture samples (left). The two tables list how wall textures were combined and which arrangements were rewarded. Each row within a table corresponds to a unique discrimination and vertical bars (i.e. |) indicate spatial arrangements within each building (‘X|Y' indicates ‘X' to the left of ‘Y'). The ‘+' column denotes wall texture combinations that were rewarded while the ‘−' column denotes wall texture combinations that were unrewarded. Note: The reward was equally likely to be in the left or right building. Lower: Example of a trained discrimination within the virtual‐reality reality environment. [Color figure can be viewed at wileyonlinelibrary.com]

Three discriminations were learnt (see Fig. 1). In each discrimination there were two buildings and each building comprised two textures (in other words, each building was a “compound” stimulus). Participants were required to learn not only which combination of textures was rewarded but also which spatial arrangement of textures was rewarded. For example, when texture A appeared with texture B, the building with A to the left of B was rewarded (A|B). However, when texture A appeared with texture C, the building with C to the left of A was rewarded (C|A). This form of “structural” configural learning, which includes a spatial or temporal component, is thought to depend on hippocampal learning mechanisms (Sanderson et al., 2006; Aggleton et al., 2007). There were 48 trials per discrimination which were presented in a pseudorandom order.

The initial learning phase also included a set of non‐memory discriminations (location of gold visible at trial onset) and a further set of three discriminations that constituted a transverse patterning contingency (see Moses et al., 2009). However, these trials were not subject to a generalization test after leaning and are therefore not discussed in the current study.

The generalization phase involved 12 test trials. Here each rewarded wall texture configuration was presented alongside an unrewarded configuration that had only ever been trained in the context of a different discrimination (e.g., A|B+ vs. A|C−, see Fig. 1). As such, these trials involved making novel discriminations between stimuli on the basis of an equivalence class membership (rewarded vs. unrewarded). Encoding‐based models of generalization predict that during the initial training phase, participants not only learn specific relationships between texture combinations (A|B+ vs. B|A−), but also the superordinate equivalence classes (A|B+, B|C+, and C|A+ all belong to a rewarded class whereas B|A−, C|B−, and A|C− all belonging to an unrewarded class). Given this, we would expect brain activity during the initial training phase to be correlated with subsequent generalization performance. In contrast, retrieval‐based models predict that generalization depends on recalling trained associations when stimuli are presented in a novel combination. Under this view, we would expect brain activity during the generalization test itself to correlate with generalization performance. Relatively few generalization trials were run to ensure that participants did not develop a well‐practiced response to each trial type.

Behavioral outputs from the task were binary sequences indicating correct versus incorrect responses on each trial. Trials were also coded as incorrect when no response was made within 3 s. For each discrimination of the initial learning phase, these binary sequences were then converted into learning curves using a state‐space smoothing model developed by Smith et al. (2004). This model indicates a “learning trial” defined as the first trial at which there is a significant level of certainty (*P* < 0.05) that a subject is performing above chance and the discrimination has been learnt. All participants included in the sample reached the learning trial for each discrimination at least five trials before the end of training (mean learning trial = 22.56, S.D. = 8.37).

We wished to group each trial of the experiment into one of four stages based on how many of the three discriminations had been learnt; from “stage 0” (prior to the learning trial of any discrimination), to “stage 3” (after all three discriminations had been learnt). Across participants, the mean (and S.D.) number of trials within each stage were as follows; Stage 0 = 43.33 (23.39), Stage 1 = 23.13 (22.75), Stage 2 = 21.53 (20.50), Stage 3 = 56 (33.79). Importantly, response times did not significantly differ between learning stage; *F*(3,42) = 1.125, *P* = .350. As such, it is unlikely that any BOLD effects coincident with learning stage are a result of differential reaction times. Performance on the 12 generalization trials was taken as the proportion of correct responses. Although each subject included in the analysis showed a high degree of learning on each of the directly trained discriminations, generalization performance was highly variable across subjects; mean proportion correct: 0.73, range 0.33–0.92.

Encoding‐based models of generalization propose that new representations overlap with existing ones, such that the commonalities across different discrimination pairs are also represented. We therefore looked for brain regions where encoding‐related BOLD activity increased linearly with the number of discriminations that had been learnt. This pattern of results would be consistent with the notion that the representation of one discrimination automatically triggers the retrieval of overlapping discriminations that have been learnt by that stage. As an additional and stronger test of encoding‐based models, we carried out a between‐subjects’ analysis to test whether BOLD activity during the final stage of learning (stage 3) correlated with subsequent generalization performance.

The imaging analyses were performed in SPM8 (http://www.fil.ion.ucl.ac.uk/spm, see Supporting Information for details about the imaging protocols and pre‐processing). Following image pre‐processing, first‐level models of the fMRI data were produced that specified five regressors of interest; four of these modeled the trial onsets within each learning stage (i.e., stages 0–3) and an additional regressor modeled the trial onset of each generalization test trial. Nuisance regressors included motion parameters and a vector coding for drift in the MR signal. HRF amplitude estimates relating to each event of interest were then subject to group‐level analyses.

At the group level, we specified a mixed‐effects regression model that tested for linear increases in activity across the four learning stages (random intercepts for each subject were also modeled). This revealed four clusters showing the predicted linear increases, each significant after controlling for the family‐wise error rate at the cluster‐level (whole‐brain, map‐wide height threshold was *P* < 0.001); see Table 1 (upper) and Figure 2. An unthresholded statistical image for this contrast can be viewed and downloaded at http://neurovault.org/collections/2058/. The analysis did not reveal any significant linear increases or decreases in hippocampal BOLD activity when either performing a small volume correction within an bilateral hippocampal ROI (Tzourio‐Mazoyer et al., 2002) or, averaging across all hippocampal voxels, *t*(14) = −1.285, *P* = 0.220.

**Figure 2:**
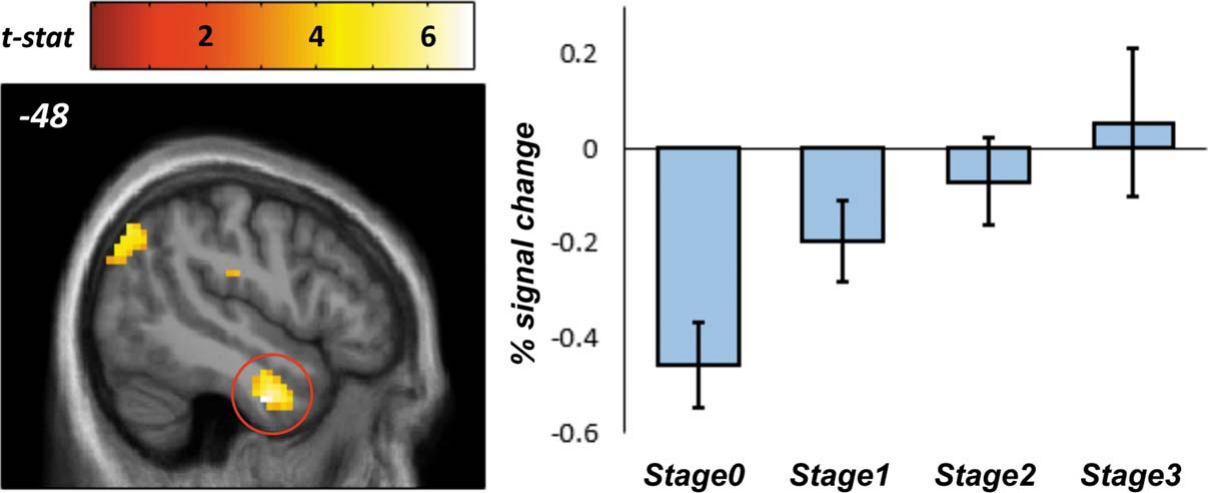
Left panel: Cluster in the left inferior temporal gyrus (ITG) exhibiting linear increases in activity as a function of the number of learnt discriminations. Right panel: Bar chart illustrating the ITG effect. Because the plot shows data selected by a whole‐brain analysis, the bars are a biased representation of the true effect size in this region. Error bars indicate 95% confidence intervals. [Color figure can be viewed at wileyonlinelibrary.com]

**Table 1:** Clusters Showing Linear Changes in BOLD Activity Across the Four Learning Stages

Region	Peak MNI [*x*, *y*, *z*]	Peak *t*	Cluster size
L Inferior temporal gyrus	[−48, −09, −33]	6.77	132
Ventromedial prefrontal (L & R)	[−15, +27, −09]	6.57	162
L insula and white matter	[−27, −57, +09]	5.87	187
L angular gyrus	[−42, −72, +45]	5.78	109

For the clusters exhibiting activation increases as a function of learning stage, we, we next examined whether BOLD activity during stage 3 (i.e., the final stage of learning) was correlated with subsequent generalization performance (across participants). A significant positive association was detected in the inferior temporal gyrus but not in any other cluster, *r*(13) = 0.666, *P* = 0.028 (Bonferroni adjusted for four comparisons). This suggests that the region may be involved in representing overlapping associative information in a manner that supports mnemonic generalizations as specified by encoding‐based models.

Retrieval‐based models of generalization propose that generalizations are carried out “on‐the‐fly” (i.e., during novel tests) when problems cannot be solved by reproducing exactly what has been learnt. We therefore carried out a between‐subjects’ analysis to identify the brain regions that were activated most by individuals who performed best on the generalization test. To examine this, the HRF amplitude estimates relating to generalization test trials were correlated with generalization performance at the second‐level. This revealed one cluster in the right posterior hippocampus showing a positive association (see Fig. 3). This effect was significant after controlling for the family‐wise error rate at the peak level within a bilateral hippocampal ROI (Tzourio‐Mazoyer et al., 2002); *r*(13) = 0.861, *P* = 0.011, peak MNI coordinate = [24,−30, −06]). The unthresholded statistical image for this contrast can be viewed and downloaded at http://neurovault.org/collections/2058/. A Kolmogorov–Smirnov test verified that the assumption of normally distributed errors had been met, *D*(15) = 0.143, *P* > 0.200. However, it is noteworthy that the performance of one subject was numerically below chance (i.e., <0.5) and that this subject yielded a relatively large regression‐residual. As such, we examined whether the hippocampal effect was robust to the removal of this data point. Even after excluding the outlier, the correlation remained significant; *r*(13) = 0.761, *P* < 0.001. No other significant effects were detected.

**Figure 3:**
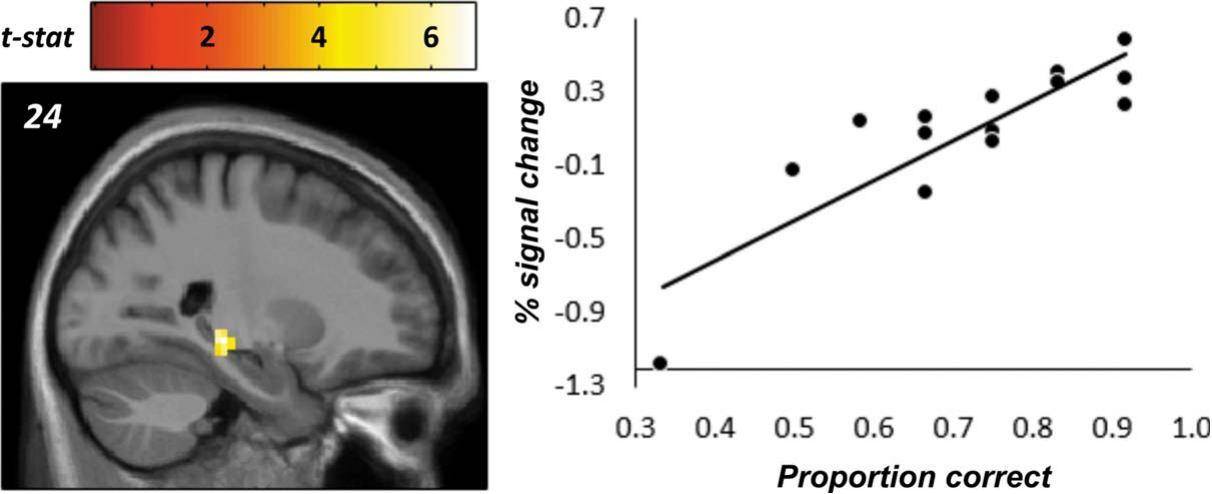
Correlation between generalization performance and right hippocampal activity during the generalization test. [Color figure can be viewed at wileyonlinelibrary.com]

We next explored whether the hippocampal region identified above showed a BOLD correlation with generalization performance during the final stage of learning (stage 3). A significant positive association was indeed found (*r* = 0.599, *P* = 0.018) suggesting that the region may also play a role in encoding‐based generalization. We further examined whether the hippocampal cluster exhibited linear changes in activity coincident with initial learning. Across the three learning stages, there was a marginally significant decrease in BOLD activity; *t*(44) = −1.98, *P* = 0.054. This latter result neither corroborates or rules out a role for the hippocampus in learning conjunctions between related discriminations, especially given that decreases in hippocampal BOLD may actually reflect successful associative binding (Olsen et al., 2012).

Given the above, an important possibility is whether the hippocampal generalization effects actually reflect the retrieval of specific reward contingencies rather than a generalization process per se. Indeed, our study was not ideally suited to examine associative generalization since participants could have solved the generalization task by retrieving the value of individual compound stimuli (e.g., A|B is rewarded). This would entail a form of “stimulus” generalization, that is, the extension of a prelearnt response across similar contexts. As such, while we did not detect any significant modulations of hippocampal BOLD over the course learning, it is possible that the hippocampal effects simply reflect better stimulus generalization in participants with the strongest representations of the original contingencies. However, we consider this to be unlikely. Participants’ ability to generalize was uncorrelated with learning rate (as measured by the time to reach stage 3); *r*(13) = 0.19, *P* = 0.498. Furthermore, the hippocampal correlations with generalization at both stage 3 and the generalization test held after controlling for learning rate; *r*(12) = 0.649, *P* = 0.013, and *r*(12) = 0.843, *P* < 0.001 (respectively). Given these findings, we suggest that the hippocampus contributed to generalization performance over and above simply representing the directly trained associations.

The main focus of this study is on the role of the hippocampus in learning a set of related discriminations and generalizing them on the basis of equivalence class membership. We found no strong evidence for learning‐related BOLD changes within the hippocampus that would be consistent with encoding‐based models of generalization. However, it remains a possibility that the small number of subjects included in our final sample may have limited our ability to detect such effects. Nonetheless, during the generalization test, one hippocampal region showed a robust correlation between BOLD activity and individuals’ ability to generalize. Importantly, the association between generalization performance and hippocampal BOLD was also present in the learning phase after all discriminations had been learnt. This suggests that representations necessary for accurate generalization may have been present in the hippocampus before there was any requirement to make generalizations. Although post‐hoc, this latter finding is partially supportive of an encoding‐based mechanism.

Taken together, the results of our study support a role for the hippocampus in both “on‐the‐fly” generalizations that are computed in novel situations and in generalizations during initial learning where the hippocampus activates information from previous events which share features relevant to the current event. Our results are therefore consistent with proposals that the hippocampus contributes to generalization at both encoding and test via the same fundamental mechanism (Zeithamova and Preston, 2010; Zeithamova et al., 2012b).

The region of the hippocampus associated with generalization in our study was midway along its length. Several neuroimaging studies have implicated the anterior hippocampus in generalization (Heckers et al., 2004; Shohamy and Wagner, 2008; Schlichting et al., 2015). However, it may be that the site of hippocampal involvement in generalization depends on the nature of the information that must be generalized. It has been argued that the longitudinal axis of the hippocampus is characterized by a functional gradient whereby mnemonic representations that are more local to each other in space or time are coded by more posterior hippocampal regions (see, Strange et al., 2014). Moreover, associations between events that are closely related within a narrative structure may be represented by posterior parts of the hippocampus while associations between more distally related events are represented by anterior regions (Collin et al., 2015). It is therefore possible that the precise locus of hippocampal involvement in generalization depends on the nature of the associations that are being generalized.

Outside of the hippocampus, learning‐related effects were observed in several regions, including two which are strongly implicated in semantic processing (the left angular gyrus [AG] and left inferior temporal gyrus [ITG]). Unlike the hippocampus, the ITG showed activation increases suggestive of gradually learning generalized memory traces. Interestingly, activity in this region also correlated with generalization performance during the initial learning phase. Damage to the ITG results in a loss of semantic knowledge (e.g., Mummery et al., 2000; Schwartz et al., 2009). Given this, we suggest that the left ITG may be responsible for storing integrated memory traces relevant to subsequent generalization. In contrast, the left AG is believed to play a role in matching the conjunctions of perceptual features to specific memory representations (especially in the light of competition from similar, overlapping representations; see Ansari, 2008).

We also observed learning‐related effects in the ventromedial prefrontal cortex (vmPFC). Previous learning and generalization studies have shown that vmPFC activity and its connectivity with the hippocampus correlates with subsequent generalization performance, both during encoding and retrieval (Zeithamova and Preston, 2010; Zeithamova et al., 2012a). Furthermore, lesions to this region impair the ability to make transitive inferences (Koscik and Tranel, 2012). One model suggests that the vmPFC acts to reactivate well‐established memories (i.e., schemas) when they are consistent with incoming information and to integrate new information into memory schemas (Van Kesteren et al., 2012). Thus the vmPFC, working in close association with the hippocampus, is able to create integrated knowledge representations that have been abstracted away from individual events that share common elements (see also, Schlichting and Preston, 2015).

To conclude, our findings support the hypothesis that the hippocampus enables generalization, primarily though retrieving stimuli that share overlapping details. This occurs both in novel situations, when there is an explicit requirement to generalize, but is also present during learning, when similar stimuli might be assigned spontaneously to a superordinate equivalence class.

## Supporting information

Supporting Information

## References

[hipo22688-bib-0001] Aggleton JP , Sanderson DJ , Pearce JM. 2007 Structural learning and the hippocampus. Hippocampus 17:723–734. 17598160 10.1002/hipo.20323

[hipo22688-bib-0002] Ansari D. 2008 Effects of development and enculturation on number representation in the brain. Nat Rev Neurosci 9:278–291. 18334999 10.1038/nrn2334

[hipo22688-bib-0003] Bunsey M , Eichenbaum H. 1996 Conservation of hippocampal memory function in rats and humans. Nature 379:255–257. 8538790 10.1038/379255a0

[hipo22688-bib-0004] Cohen NJ , Eichenbaum H. 1993 Memory, Amnesia, and the Hippocampal System. Cambridge (MA): MIT Press.

[hipo22688-bib-0005] Collin SH , Milivojevic B , Doeller CF. 2015 Memory hierarchies map onto the hippocampal long axis in humans. Nat Neurosci 18:562–1564. 26479587 10.1038/nn.4138 PMC4665212

[hipo22688-bib-0006] Eichenbaum H. 2004 Hippocampus: Cognitive processes and neural representations that underlie declarative memory. Neuron 44:109–120. 15450164 10.1016/j.neuron.2004.08.028

[hipo22688-bib-0007] Heckers S , Zalesak M , Weiss AP , Ditman T , Titone D. 2004 Hippocampal activation during transitive inference in humans. Hippocampus 14:153–162. 15098721 10.1002/hipo.10189

[hipo22688-bib-0008] Koscik TR , Tranel D. 2012 The human ventromedial prefrontal cortex is critical for transitive inference. J Cogn Neurosci 24:1191–1204. 22288395 10.1162/jocn_a_00203 PMC3626083

[hipo22688-bib-0009] Kumaran D , McClelland JL. 2012 Generalization through the recurrent interaction of episodic memories: A model of the hippocampal system. Psychol Rev 119:573–616. 22775499 10.1037/a0028681 PMC3444305

[hipo22688-bib-0010] McClelland JL , McNaughton BL , O'Reilly RC. 1995 Why there are complementary learning systems in the hippocampus and neocortex: Insights from the successes and failures of connectionist models of learning and memory. Psychol Rev 102:419–457. 7624455 10.1037/0033-295X.102.3.419

[hipo22688-bib-0011] Moses SN , Ryan JD , Bardouille T , Kovacevic N , Hanlon FM , McIntosh AR. 2009 Semantic information alters neural activation during transverse patterning performance. NeuroImage 46:863–873. 19281852 10.1016/j.neuroimage.2009.02.042 PMC2789295

[hipo22688-bib-0012] Mummery CJ , Patterson K , Price CJ , Ashburner J , Frackowiak RSJ , Hodges JR. 2000 A voxel‐based morphometry study of semantic dementia: Relationship between temporal lobe atrophy and semantic memory. Ann Neurol 47:36–45. 10632099

[hipo22688-bib-0013] Olsen RK , Moses SN , Riggs L , Ryan JD. 2012 The hippocampus supports multiple cognitive processes through relational binding and comparison. Front Hum Neurosci 6:146. 22661938 10.3389/fnhum.2012.00146 PMC3363343

[hipo22688-bib-0014] Preston AR , Shrager Y , Dudukovic NM , Gabrieli JD. 2004 Hippocampal contribution to the novel use of relational information in declarative memory. Hippocampus 14:148–152. 15098720 10.1002/hipo.20009

[hipo22688-bib-0015] Sanderson DJ , Pearce JM , Kyd RJ , Aggleton JP. 2006 The importance of the rat hippocampus for learning the structure of visual arrays. Eur J Neurosci 24:1781–1788. 17004941 10.1111/j.1460-9568.2006.05035.x

[hipo22688-bib-0016] Schlichting ML , Preston AR. 2015 Memory integration: Neural mechanisms and implications for behavior. Curr Opin Behav Sci 1:1–8. 25750931 10.1016/j.cobeha.2014.07.005 PMC4346341

[hipo22688-bib-0017] Schlichting ML , Mumford JA , Preston AR. 2015 Learning‐related representational changes reveal dissociable integration and separation signatures in the hippocampus and prefrontal cortex. Nat Commun 6:1–10. 10.1038/ncomms9151 PMC4560815 26303198

[hipo22688-bib-0018] Schwartz MF , Kimberg DY , Walker GM , Faseyitan O , Brecher A , Dell GS , Coslett HB. 2009 Anterior temporal involvement in semantic word retrieval: Voxel‐based lesion‐symptom mapping evidence from aphasia. Brain 132:3411–3427. 19942676 10.1093/brain/awp284 PMC2792374

[hipo22688-bib-0019] Shohamy D , Wagner AD. 2008 Integrating memories in the human brain: Hippocampal‐midbrain encoding of overlapping events. Neuron 60:378–389. 18957228 10.1016/j.neuron.2008.09.023 PMC2628634

[hipo22688-bib-0020] Smith AC , Frank LM , Wirth S , Yanike M , Hu D , Kubota Y , Graybiel AM , Suzuki WA , Brown EN. 2004 Dynamical analysis of learning in behavior experiments. J Neurosci 24:447–461. 14724243 10.1523/JNEUROSCI.2908-03.2004 PMC6729979

[hipo22688-bib-0021] Strange BA , Witter MP , Lein ES , Moser EI. 2014 Functional organization of the hippocampal longitudinal axis. Nat Rev Neurosci 15:655–669. 25234264 10.1038/nrn3785

[hipo22688-bib-0022] Tzourio‐Mazoyer N , Landeau B , Papathanassiou D , Crivello F , Etard O , Delcroix N , … Joliot M. 2002 Automated anatomical labeling of activations in SPM using a macroscopic anatomical parcellation of the MNI MRI single‐subject brain. NeuroImage 15:273–289. 11771995 10.1006/nimg.2001.0978

[hipo22688-bib-0023] van Kesteren MT , Ruiter DJ , Fernández G , Henson RN. 2012 How schema and novelty augment memory formation. Trends Neurosci 35:211–219. 22398180 10.1016/j.tins.2012.02.001

[hipo22688-bib-0024] Zeithamova D , Preston AR. 2010 Flexible memories: Differential roles for medial temporal lobe and prefrontal cortex in cross‐episode binding. J Neurosci 30:14676–14684. 21048124 10.1523/JNEUROSCI.3250-10.2010 PMC6633616

[hipo22688-bib-0025] Zeithamova D , Dominick AL , Preston AR. 2012a Hippocampal and ventral medial prefrontal activation during retrieval‐mediated learning supports novel inference. Neuron 75:168–179. 22794270 10.1016/j.neuron.2012.05.010 PMC3398403

[hipo22688-bib-0026] Zeithamova D , Schlichting ML , Preston AR. 2012b The hippocampus and inferential reasoning: Building memories to navigate future decisions. Front Hum Neurosci 6:1–14. 22470333 10.3389/fnhum.2012.00070 PMC3312239

