# Supplementary material for: The role of the hippocampus in generalizing configural relationships
Source: Hippocampus. 2017 Jan 24;27(3):223–8. doi: 10.1002/hipo.22688 (PMC5324609; doi:10.1002/hipo.22688)
Supplement: Supplementary file 1 — Supporting Information [file HIPO-27-223-s001.pdf]

Supplementary methods

MRI Acquisition

All images were acquired in a 1.5T Siemens Avanto scanner equipped with a 32-channel phased array head coil. During the task, gradient-echo T2*-weighted scans were acquired using echo-planar imaging (EPI) recording 34 axial slices (approximately 30° to AC-PC line; ascending interleaved) and the following parameters; TR = 2520 ms, TE = 43 ms, flip angle (FA) = 90°, slice thickness = 3 mm, inter-slice gap = 0.6 mm in-plane resolution = 3 x 3 mm, & acquisition matrix = 64 x 64. To allow for T1 equilibrium, the first 5 EPI volumes were acquired before the task started and then discarded. A field map scan was also acquired to correct inhomogeneity-based distortions (see Image Preprocessing section below). Finally, for purposes of coregistration and image normalization, a whole-brain T1-weighted structural scan was acquired with a $1mm^3$ resolution using an MP-RAGE pulse sequence.

Image Preprocessing

Image preprocessing was performed in SPM8 (www.fil.ion.ucl.ac.uk/spm) and using custom written code in MATLAB. First, each subject's EPI volumes were corrected for inter-slice acquisition delay and spatially realigned to the first image in the time series. At the same time, images were corrected for field inhomogeneity based geometric distortions (as well as the interaction between motion and such distortions) using the Realign and Unwarp algorithms in SPM (Andersson et al., 2003; Hutton et al., 2002). EPI time series data were warped to MNI space with transformation parameters derived from the structural scans (using the DARTEL toolbox; Ashburner, 2007). Subsequently, the EPI volumes were spatially smoothed with an isotropic 8 mm FWHM Gaussian kernel prior to GLM analysis. BOLD activations in figures 2 and 3 are displayed over a group mean structural image in MNI space.

References

Andersson JL, Skare S, Ashburner J. 2003. How to correct susceptibility distortions in spin-echo echo-planar images: application to diffusion tensor imaging. *NeuroImage, 20*(2), 870-888.

Ashburner J. 2007. A fast diffeomorphic image registration algorithm. *NeuroImage 38*(1):95- 113.

Hutton C, Bork A, Josephs O, Deichmann R, Ashburner J, Turner R. 2002. Image distortion correction in fMRI: a quantitative evaluation. *NeuroImage 16*(1):217-240.
